# Supplementary material for: The impact of tumor microenvironment and treatment schedule on the effectiveness of radiation therapy
Source: PLoS One. 2025 Sep 17;20(9):e0331509. doi: 10.1371/journal.pone.0331509 (PMC12443294; doi:10.1371/journal.pone.0331509)
Supplement: S1 Text — (PDF) [file pone.0331509.s001.pdf]

# Supplementary Text

**S1 Text: Sensitivity analysis on the transition rate $k_M$, the clearance rate $C_r$ and the parameter $\alpha$ of the Linear Quadratic model.**

A sensitivity analysis was performed on the transition rate $k_M$, the clearance rate $C_r$ and the parameter $\alpha$ of the Linear Quadratic model, to evaluate their influence on the model outputs. The parameters were varied at five levels, 1.1, 1.05, 1, 0.95 and 0.9 times their baseline values as given in Table 2. From the simulations the total variance of tumor volume was calculated for each parameter. The total variance of tumor volume was given by the following equation,

$$Tot.Var. = \sum_{i}^{5} 1/T \int_{0}^{T} \left(V_i(t) - \bar{V}(t)\right)^2 dt \quad (1)$$

where the integration is performed over time $(t)$ for $i$ -th different levels of each parameter, $T$ is the time interval of integration, $V_i$ is the volume of $i$-th level at current time $(t)$, $\bar{V}$ is the average volume of the five different levels at current time $(t)$, and finally the sum of the variance of the five levels is calculated. The results of the sensitivity analysis are presented in **S1 Table** and in **S11-S13 Figs**. Since both $C_r$ and $\alpha$ become active on the first day of treatment (Day 10); therefore, total variance in tumor volume attributable to each parameter was calculated from Day 10 through the final measurement day. As shown in **S1 Table**, the transition rate $k_M$ exhibits the highest sensitivity, indicating it has the greatest impact on model outputs. Regarding the other two parameters, $\alpha$ shows a slightly higher contribution to the variance than $C_r$, indicating a modestly greater effect on the model's tumor volume predictions.
