# Supplementary material for: The impact of tumor microenvironment and treatment schedule on the effectiveness of radiation therapy
Source: PLoS One. 2025 Sep 17;20(9):e0331509. doi: 10.1371/journal.pone.0331509 (PMC12443294; doi:10.1371/journal.pone.0331509)
Supplement: S1 Table — (PDF) [file pone.0331509.s002.pdf]

Supplementary Tables

S1 Table: The logarithm of total variance in tumor volume for the parameters of the sensitivity analysis (log($\boldsymbol{Tot.Var.}$)>1)

Parameter	log($\boldsymbol{Tot.Var.}$)
$\boldsymbol{k_M}$	4.29
$\boldsymbol{C_r}$	2.27
$\boldsymbol{a}$	2.50
