# Supplementary figures and images for: The impact of tumor microenvironment and treatment schedule on the effectiveness of radiation therapy
Source: PLoS One. 2025 Sep 17;20(9):e0331509. doi: 10.1371/journal.pone.0331509 (PMC12443294; doi:10.1371/journal.pone.0331509)

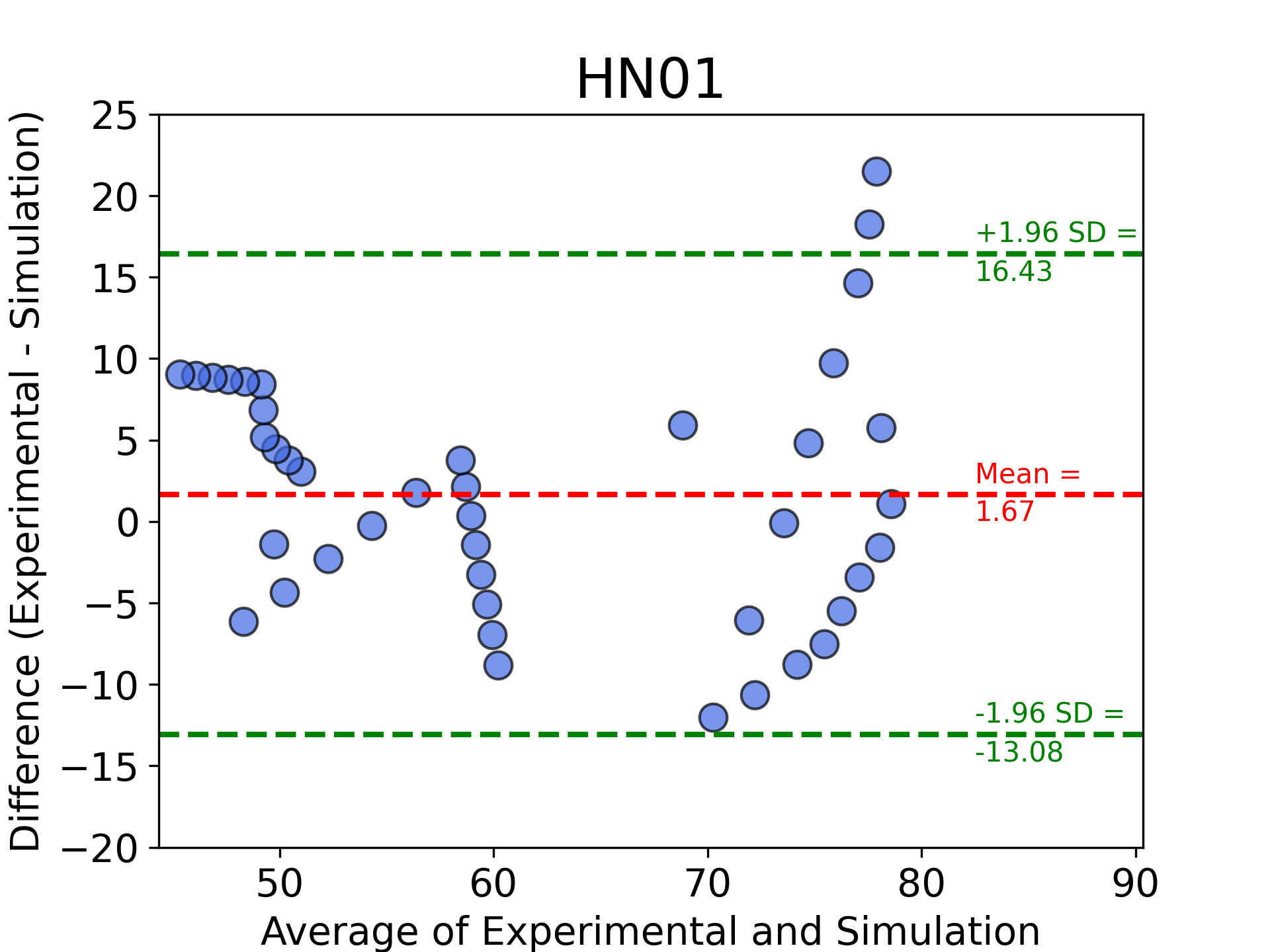

Supplement: S1 Fig — Each dot represents the difference versus the mean of the two measurements for a single time point. (TIF) [file pone.0331509.s003.tif]

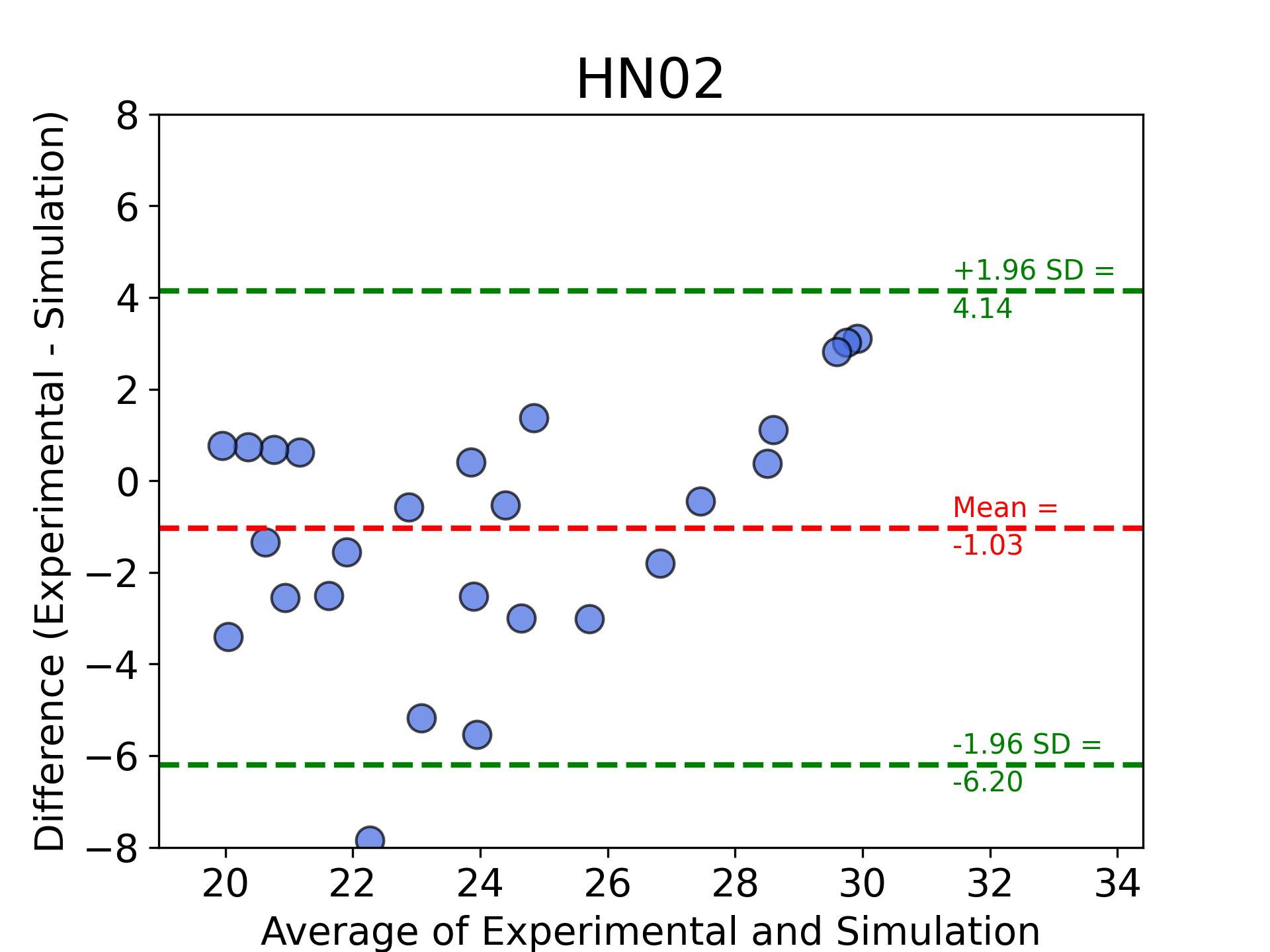

Supplement: S2 Fig — Each dot represents the difference versus the mean of the two measurements for a single time point. (TIF) [file pone.0331509.s004.tif]

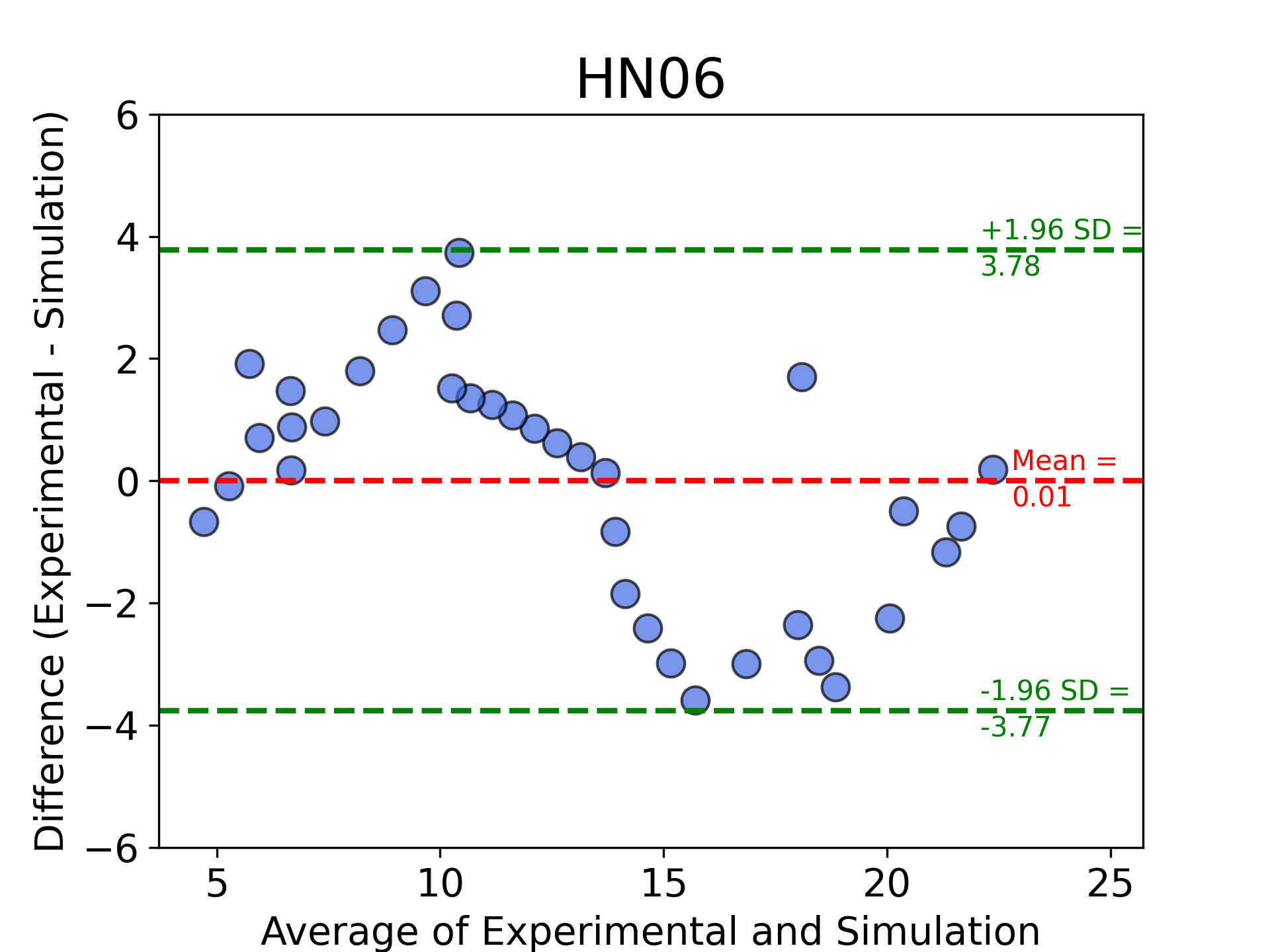

Supplement: S4 Fig — Each dot represents the difference versus the mean of the two measurements for a single time point. (TIF) [file pone.0331509.s006.tif]

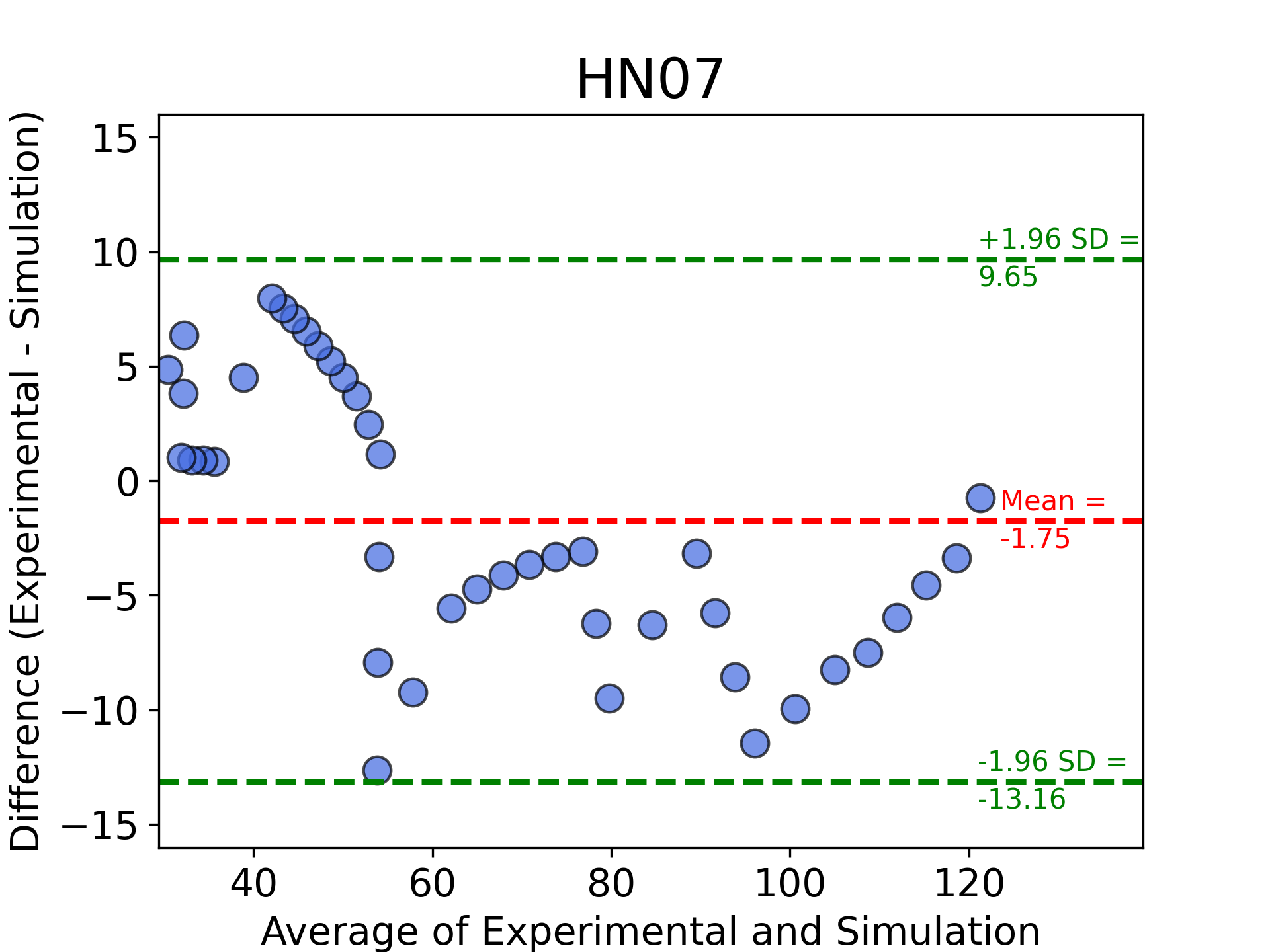

Supplement: S5 Fig — Each dot represents the difference versus the mean of the two measurements for a single time point. (TIF) [file pone.0331509.s007.tif]

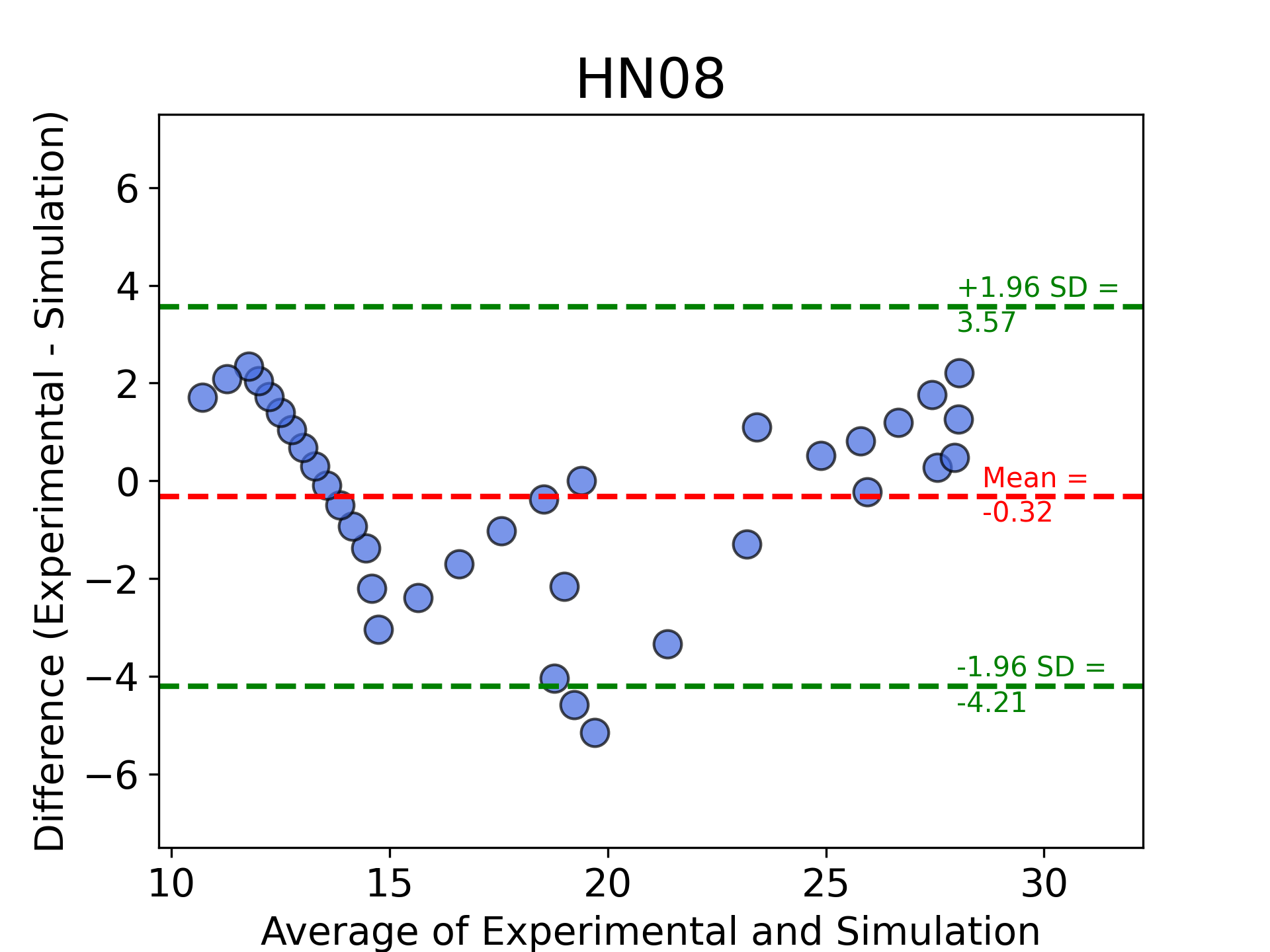

Supplement: S6 Fig — Each dot represents the difference versus the mean of the two measurements for a single time point. (TIF) [file pone.0331509.s008.tif]

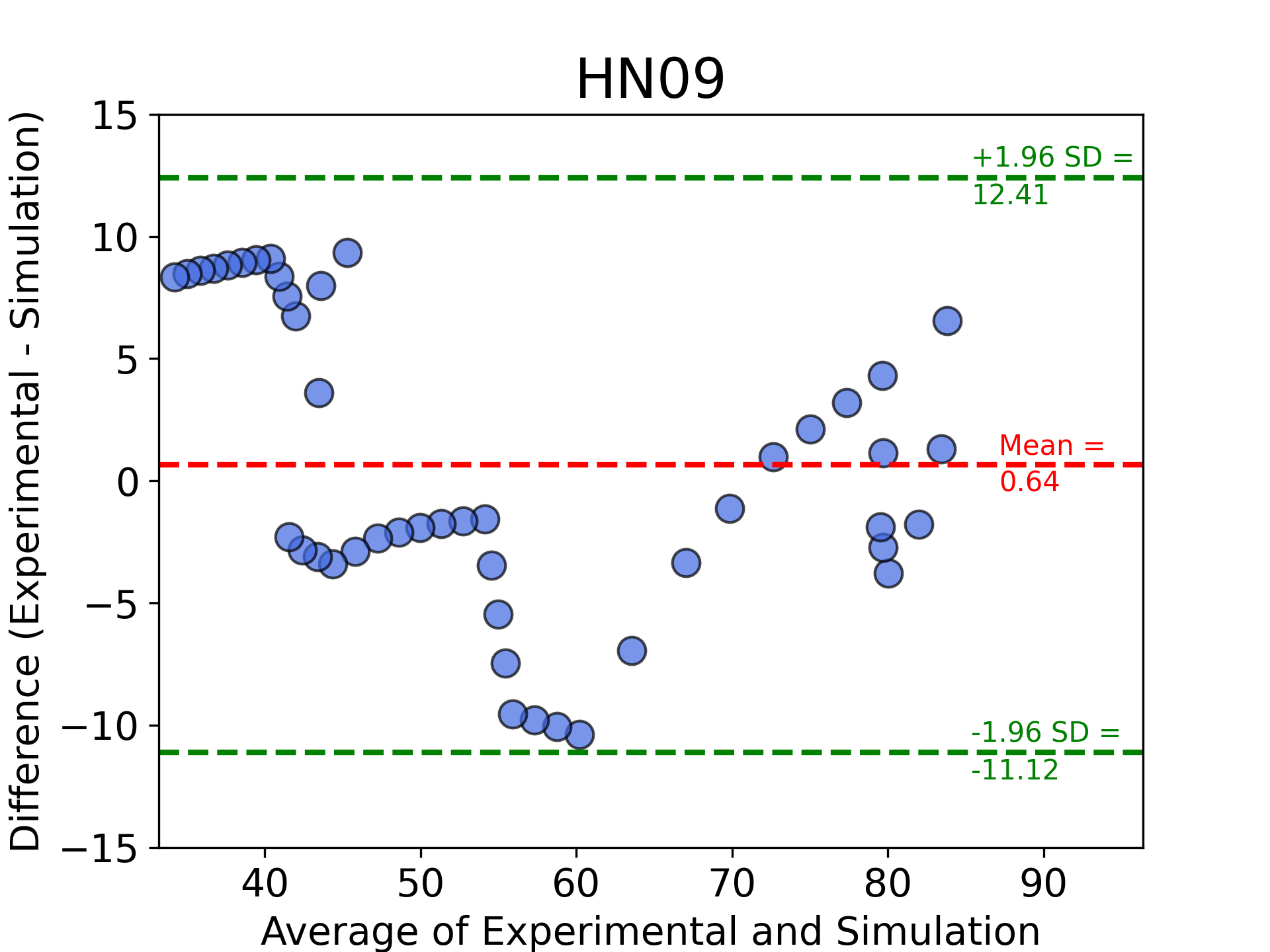

Supplement: S7 Fig — Each dot represents the difference versus the mean of the two measurements for a single time point. (TIF) [file pone.0331509.s009.tif]

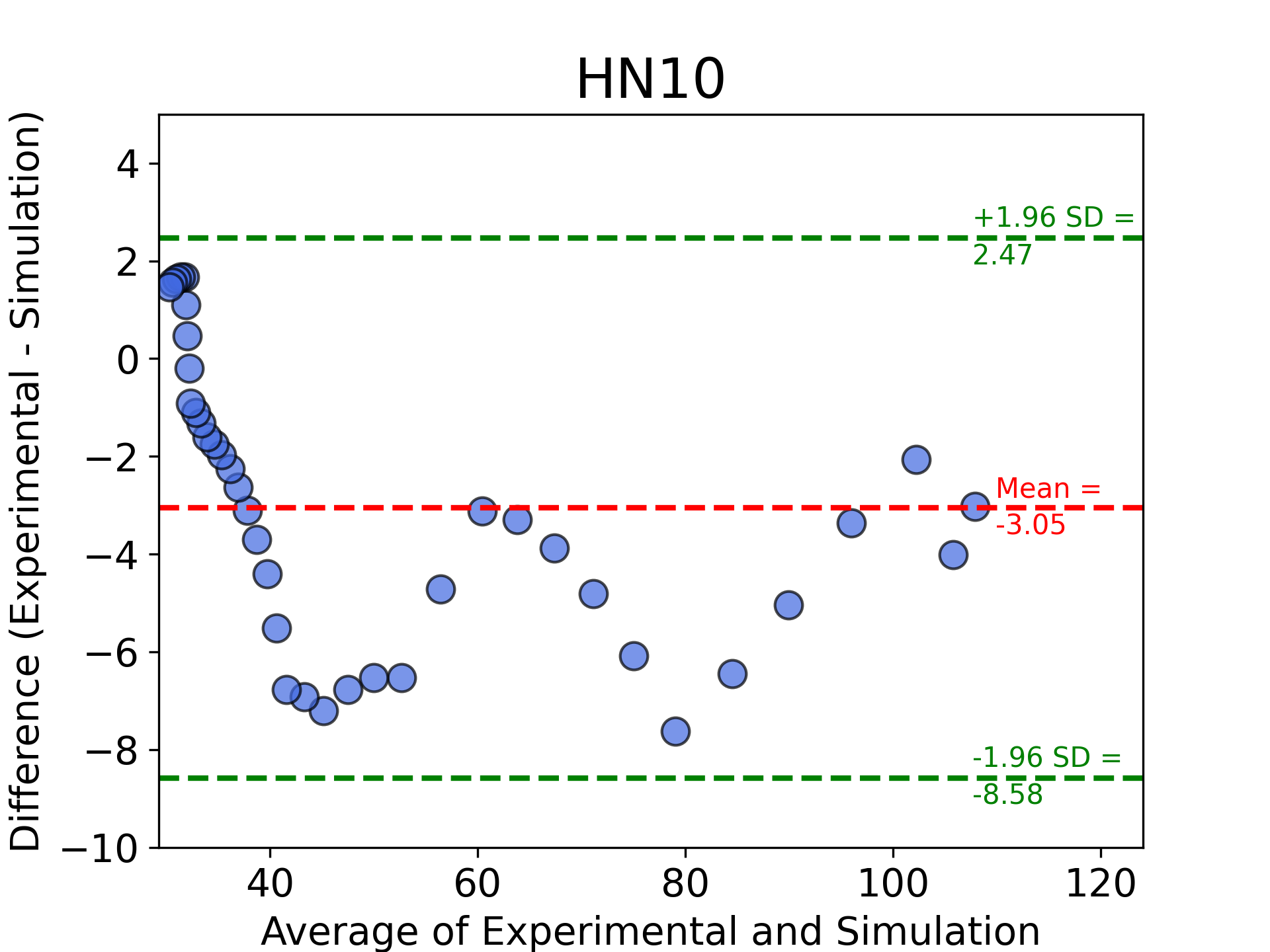

Supplement: S8 Fig — Each dot represents the difference versus the mean of the two measurements for a single time point. (TIF) [file pone.0331509.s010.tif]

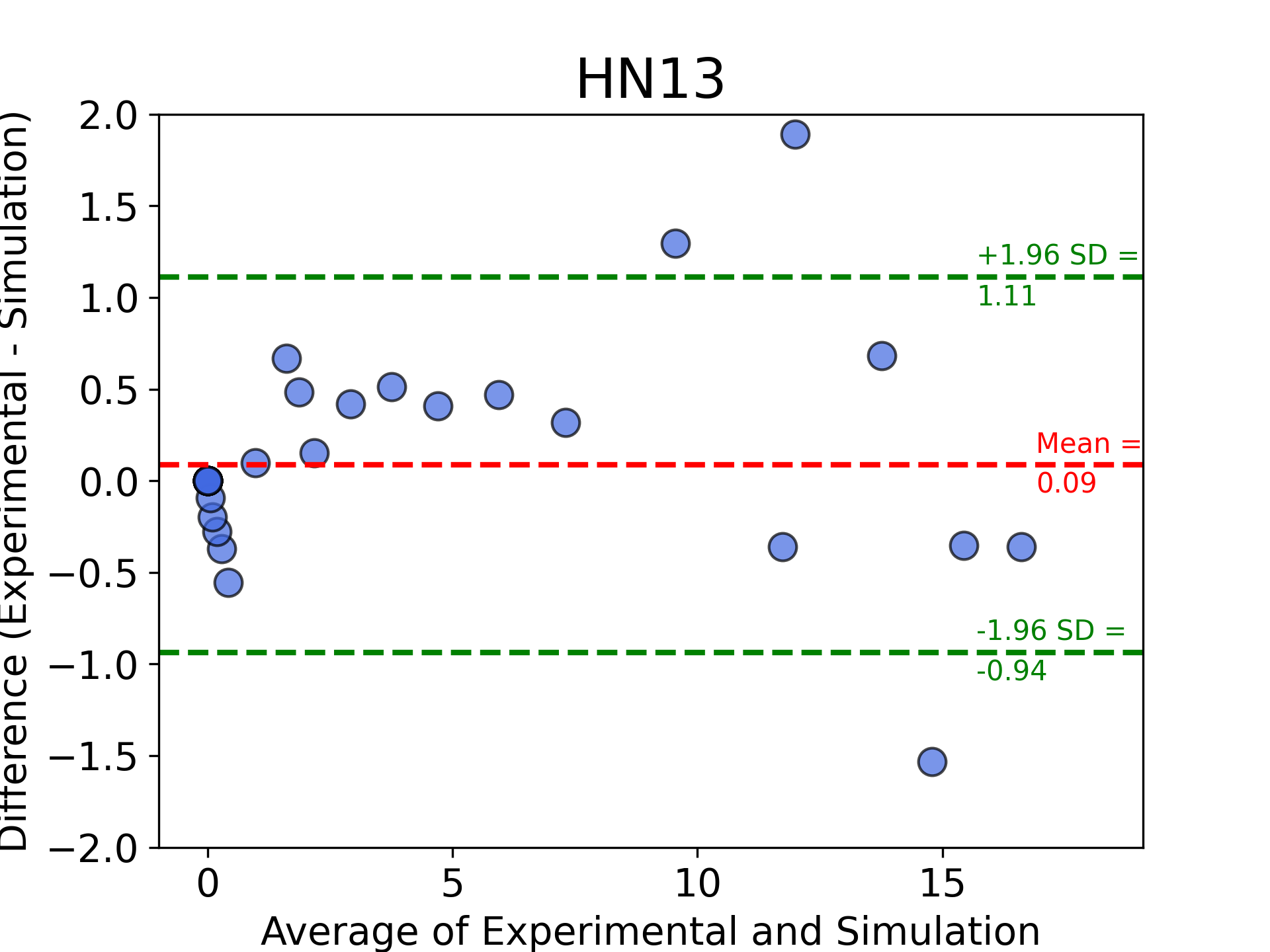

Supplement: S9 Fig — Each dot represents the difference versus the mean of the two measurements for a single time point. (TIF) [file pone.0331509.s011.tif]

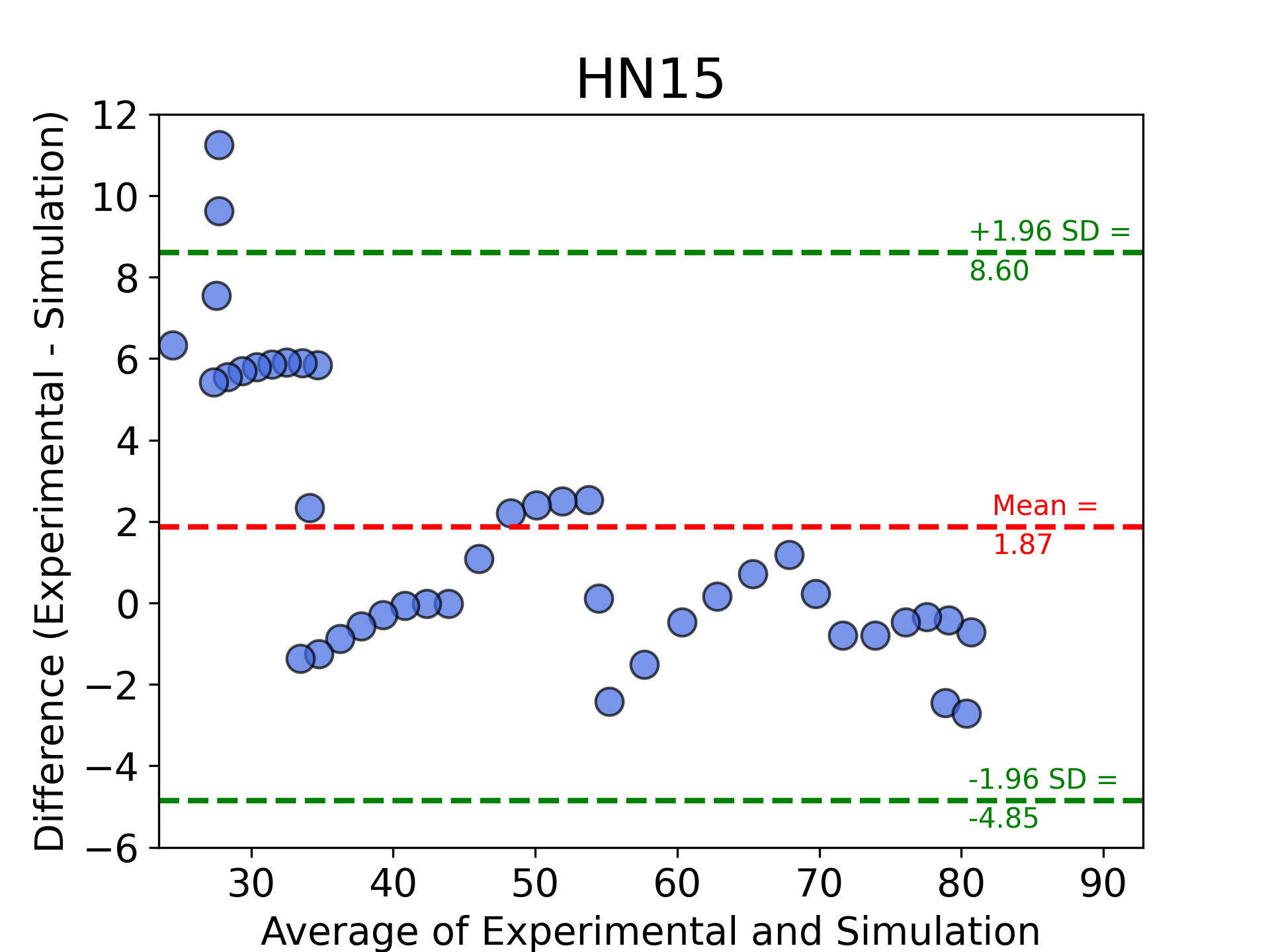

Supplement: S10 Fig — Each dot represents the difference versus the mean of the two measurements for a single time point. (TIF) [file pone.0331509.s012.tif]

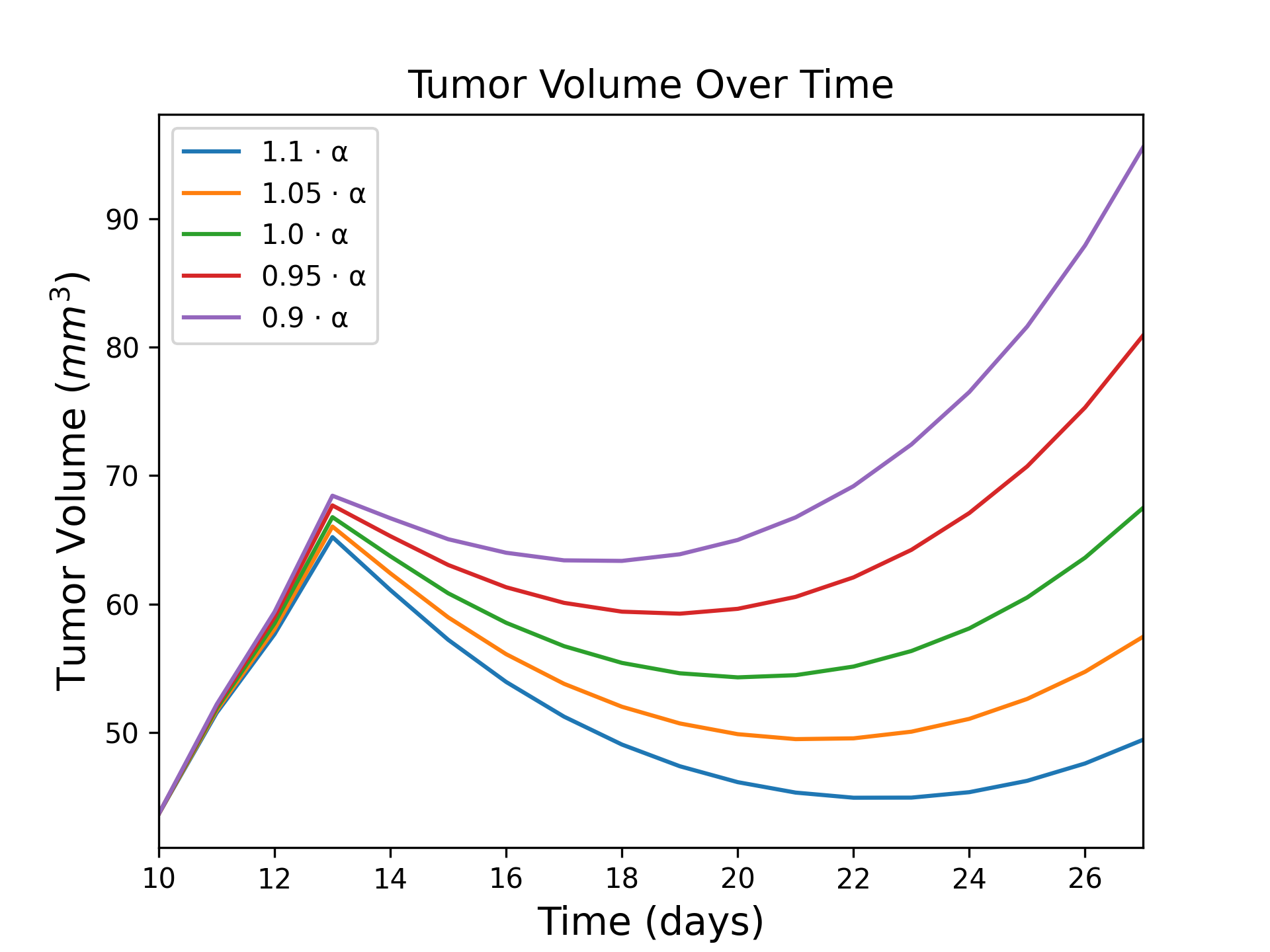

Supplement: S11 Fig — The parameter varied at five levels, 1.1, 1.05, 1, 0.95 and 0.9 times its baseline value as given in Table 2. (TIF) [file pone.0331509.s013.tif]

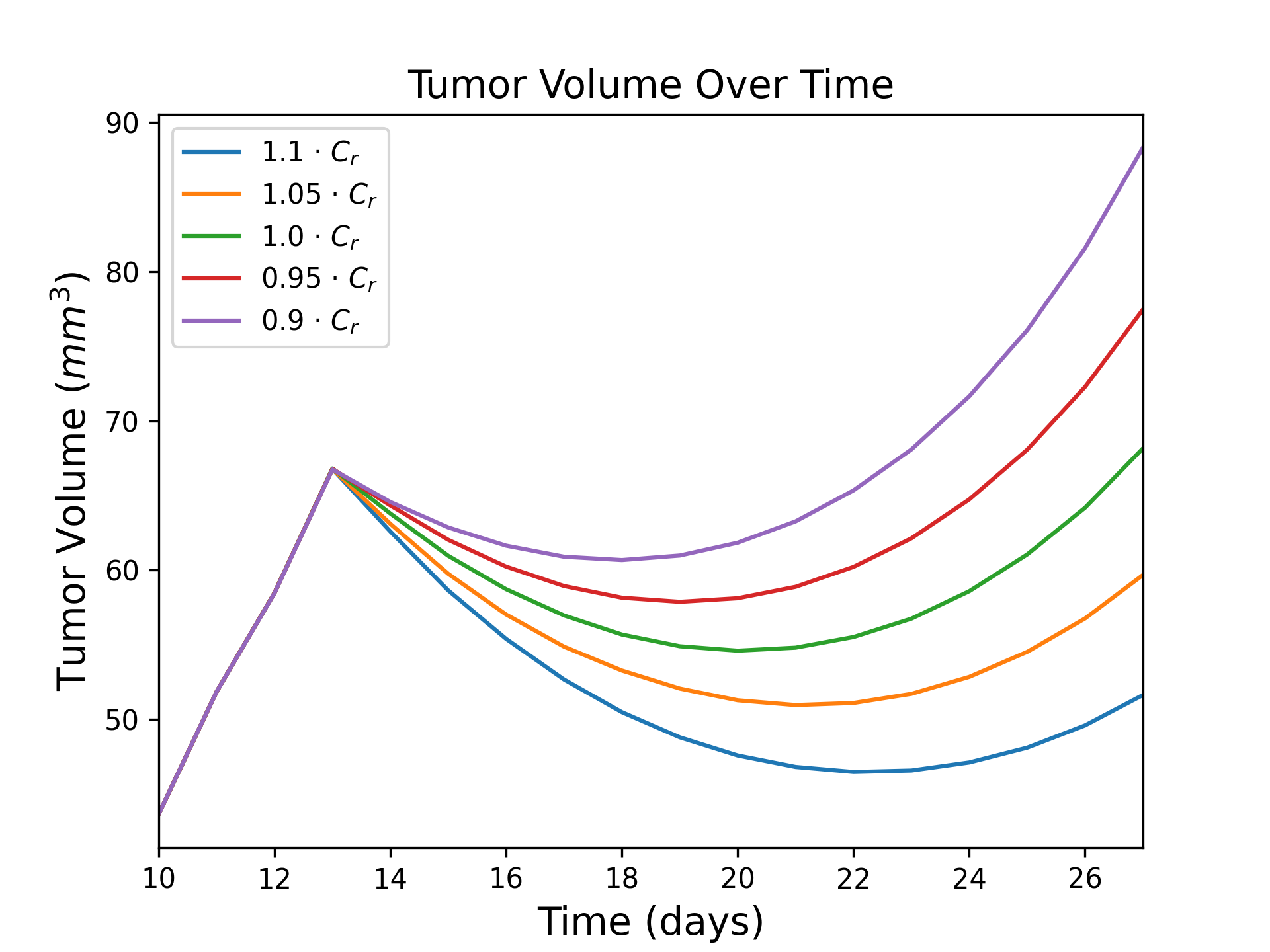

Supplement: S12 Fig — The parameter varied at five levels, 1.1, 1.05, 1, 0.95 and 0.9 times its baseline value as given in Table 2. (TIF) [file pone.0331509.s014.tif]

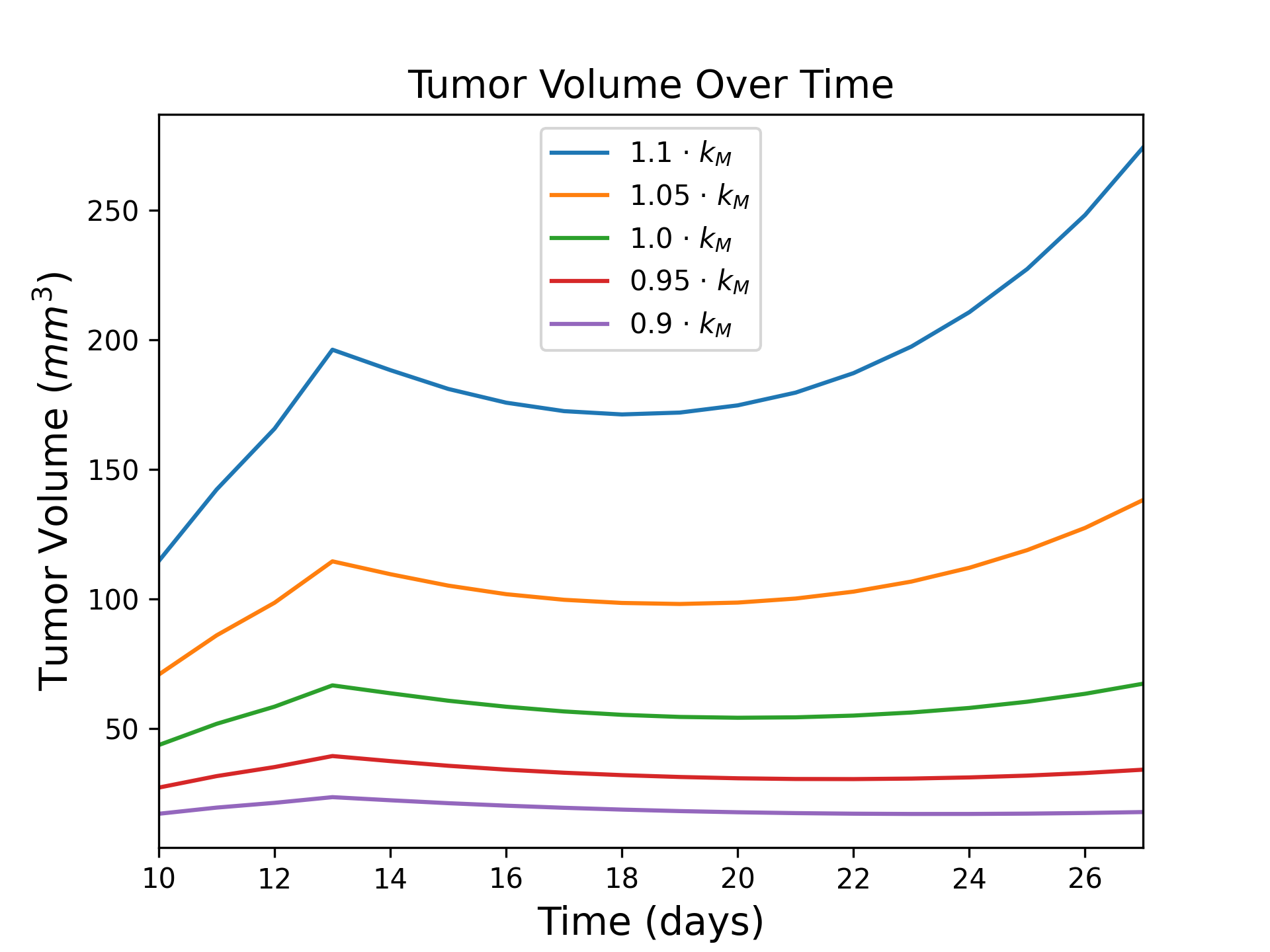

Supplement: S13 Fig — The parameter varied at five levels, 1.1, 1.05, 1, 0.95 and 0.9 times its baseline value as given in Table 2. (TIF) [file pone.0331509.s015.tif]
