# Supplementary material for: The impact of tumor microenvironment and treatment schedule on the effectiveness of radiation therapy
Source: PLoS One. 2025 Sep 17;20(9):e0331509. doi: 10.1371/journal.pone.0331509 (PMC12443294; doi:10.1371/journal.pone.0331509)
Supplement: Attachment — Submitted filename: Response to Reviewers.docx [file pone.0331509.s016.docx]

# Response to Reviewers

We thank all the reviewers for the constructive comments and helpful suggestions, which have helped us revise and strengthen our manuscript.

Please find below our point-by-point response (plain font) to the reviewers’ comments (italic). We have marked edits in the revised manuscript and supporting information files in red font (color).

# Reviewer #1

1. *The study is clear written, even if the clinical application of this model is not immediate. I suggest to better explain how parameters for tumor perfusion and oxygenation were obtained. Figures should be inserted in the text and linked to the corresponding legend.*

We thank the Reviewer for the positive evaluation of our article and the constructive comments.

We appreciate the reviewer’s suggestion. In the revised manuscript, we edited the subsection “Optimal Treatment Schedule for Prostate Cancer” of the “Results” (page 24) to clarify how functional vascular density, which is linked to tumor perfusion and oxygenation, is defined and modified. In addition, the section “Effect of tumor perfusion/oxygenation on treatment efficacy” (pages 15-17) is devoted to this topic.

Regarding the inclusion of figures in the manuscript, the PLOS One guidelines state: “Please remove your figures from within your manuscript file, leaving only the individual TIFF image files. These will be automatically included in the reviewer’s PDF.” In order to comply with these guidelines, we have excluded the figures from the manuscript text.

# Reviewer #2

*The manuscript explores the relationship between tumour microenvironment factors (oxygenation, perfusion) and different radiotherapy schedules using a mathematical modelling approach. The study is methodologically rigorous and provides valuable insights into optimising radiotherapy strategies. The validation using preclinical and clinical datasets enhances the credibility of the model. However, there are limitations concerning model assumptions, generalizability, and the lack of toxicity considerations.*

*The study presents a well-structured, continuous mathematical model that incorporates tumour perfusion, oxygenation, and radiosensitivity parameters, providing a quantitative framework for understanding radiotherapy efficacy. The model is validated against murine and human clinical data, strengthening its reliability and applicability. The manuscript effectively compares conventional fractionation (CFRT), moderate hypofractionation (MHRT), and stereotactic body radiotherapy (SBRT), offering clinically relevant insights. The study demonstrates that higher vascular density improves radiotherapy effectiveness, particularly in radioresistant tumours, which is a valuable mechanistic insight. However, several issues require further attention.*

We thank the Reviewer for the overall positive evaluation of the manuscript and the constructive comments.

1. *While the model includes oxygenation effects, it does not fully incorporate tumour heterogeneity, immune response, or dynamic hypoxia variations. A discussion of these limitations should be added.*

The reviewer is right. We revised the “Discussion” section (pages 29-30) and explicitly mentioned the limitations regarding tumour heterogeneity and immune response. Regarding dynamic hypoxia variations, our model accounts for changes in oxygen concentration (Equation 10) and incorporates these variations during cell phase transitions through parameters $\tilde{a}$ and $\tilde{b}$, as well as in the calculation of the Oxygen Enhancement Ratio $OER$ (Equation 21). Additionally, the parametric analysis of the functional vascular density $S_{v}$ is directly related to dynamic hypoxia variations.

2. *The model was validated using a small dataset (10 head-and-neck cancer patients), which may limit the generalizability of the findings to a broader population or other cancer types.*

We appreciate the reviewer’s comment. In addition to the validation using data from 10 head-and-neck cancer patients, the model was initially validated against two independent murine studies from the literature. The first study involved murine HPV-positive head and neck tumors, comprising one control group and one treatment group. The second study focused on murine prostate cancer models, including one control group and four treatment groups. The agreement between the model predictions and the preclinical data is demonstrated in the paper, with the goodness of fit quantified by the coefficient of determination $R^{2}$.

In general, clinical studies involving radiotherapy for cancer treatment rarely incorporate tumor volume measurements during the course of treatment and that is why we did not include more data. This is primarily because volumetric assessment during radiotherapy is not part of routine clinical practice. For instance, in the case of prostate cancer, radiation is typically delivered to the entire gland due to the difficulty in precisely localizing malignant foci. As a result, a reduction in organ volume is not expected, and volumetric changes are not considered a meaningful indicator of treatment response. On the contrary, the prostate may even increase in size during therapy due to radiation-induced tissue damage and the subsequent development of edema. Furthermore, treatment efficacy is usually evaluated indirectly by monitoring prostate-specific antigen (PSA) levels, rather than by directly measuring changes in tumor volume.

3. *Although the model indicates that SBRT achieves the fastest tumour control, it does not account for the potential increase in normal tissue toxicity, which is a critical factor in treatment planning.*

We appreciate the reviewer’s comment. We explicitly mentioned this limitation of our model in the “Discussion” section (page 30) of the revised manuscript.

4. *The manuscript states that all relevant data are available; however, the full computational implementation or simulation parameters necessary for replication and validation by other researchers are not shared, which may hinder reproducibility.*

The reviewer is right. We added a “Data Availability” section (page 31) stating that the full COMSOL Multiphysics model report is included in the Supporting Information. It contains comprehensive simulation details necessary for replication or further analysis. Regarding the simulation parameters, Table 1 lists the general parameters that were kept constant across all simulations, while Tables 2 through 6 present the specific parameters that were varied to simulate the head and neck and prostate cancer cases. We also added in the revised “Methods” a new section “Model Description” (page 7) to provide more information about the implementation of the model.

5. *General suggestions: Consider simplifying the descriptions of key equations or adding explanatory figures, ensure that all figures clearly indicate variables and treatment groups for easier interpretation and the practical implications for radiation oncologists could be expanded.*

We appreciate the reviewer’s suggestion. All transition rates from Equations 1–5 have been incorporated into Figure 1 to enhance the clarity and interpretability of the equations.

*The manuscript is scientifically rigorous and methodologically sound, making it suitable for publication. However, key revisions are necessary. The discussion on model limitations, including tumour heterogeneity and immune response, should be expanded to better reflect the complexity of tumour behavior.* *Clinical validation should be strengthened by discussing external datasets and how the findings apply to different cancer types. The omission of toxicity analysis should be addressed, and future iterations of the model should consider integrating this aspect. To improve reproducibility, the manuscript should include computational code or a more detailed methodology. Finally, figures, tables, and explanations of the mathematical modelling should be clarified to enhance accessibility for a wider audience.*

We thank the Reviewer for the positive evaluation of our article. Additional model limitations have been included in the “Discussion” section (pages 29-30), specifically addressing tumor heterogeneity and the absence of an immune response component. Beyond the clinical validation using data from 10 head and neck cancer patients, we have further validated the model against two independent murine studies from the literature: one involving HPV-positive head and neck tumors and another involving prostate tumors. As noted previously, clinical studies involving radiotherapy for cancer treatment rarely include tumor volume measurements during the course of therapy, as this is not part of routine clinical practice. Furthermore, the lack of toxicity analysis has also been acknowledged and discussed (page 30). A new “Data Availability” (page 31) section has been added, stating that the full COMSOL Multiphysics model report is included in the Supporting Information. It contains comprehensive simulation details necessary for replication or further analysis. Finally, all transition rates from Equations 1–5 have been incorporated into Figure 1 to improve the clarity and interpretability of the methodology.

# Reviewer #3

*I appreciate the authors for their efforts in developing a modeling approach that, despite the limited data available in the literature, has the potential to contribute significantly to the personalization of radiotherapy. The concept appears promising and beneficial. I would like to present my suggestions under the following headings:*

*******Introduction****:*

*In terms of dose definitions, conventional dose is defined as 1.8–2 Gy per fraction. It may not be entirely accurate to classify all doses below 2 Gy per fraction as hyperfractionated radiotherapy.*

We thank the reviewer for this important clarification. We have revised the manuscript accordingly to more accurately define hyperfractionated radiotherapy (<1.8–2 Gy per fraction).

*******Methods***

*There is some ambiguity regarding the selection criteria for the 10 patients chosen from reference (16). It would be appropriate to clarify the exclusion criteria and the rationale behind excluding certain patients.*

We appreciate the reviewer’s insightful comment. The clinical study used to validate our model included 15 patients with newly diagnosed head and neck cancer. One patient (HN12) was deemed ineligible for the study, and the remaining 14 patients received radiotherapy at varying doses (72 Gy in 42 fractions, 70 Gy in 35 fractions, 60 Gy in 30 fractions, and 70 Gy in 32 fractions). Of these, only 13 patients were included in the published graph titled “Primary Tumor Response to RT,” which served as the basis for our validation (HN11 was not represented in the graph). We excluded patients HN03, HN04, and HN14 from our analysis because their tumor response curves overlapped in the graph, making it difficult to clearly identify and differentiate individual patient data.

*When examining the values in Figures 5 and 6, it appears that patients receiving a hyperfractionated regimen in reference (16) had lower values (R2). This suggests that the model may have a weaker correlation with patients undergoing hyperfractionated radiotherapy.*

We thank the reviewer for the constructive comment. We have revised the “Results” section (page 21), to address this point.

*The effects of potential influential factors have been outlined methodologically under separate headings. However, introducing a distinct section explaining the primary model in detail would make the study more reader-friendly. Furthermore, presenting the modeling results for all patients in the Results section in a tabular or visual format could enhance clarity.*

We appreciate the reviewer’s suggestion. We have added in the revised “Methods” a new section “Model Description” (page 7) that provides an overall description of the model in a more reader-friendly and accessible manner. In addition, Figure 1 has been revised to include all transition rates from Equations 1–5, thereby improving the clarity and interpretability of the model dynamics. Regarding the presentation of modeling results for all patients in the “Results” section, we have moved the validation curves for all patients, as well as those based on preclinical data, into the “Results” section

*Has the model validation been tested outside of head and neck cancer cases? Is it methodologically appropriate to apply a similar model to prostate cancer without any validation in prostate cancer patients? These are critical methodological questions that need to be addressed.*

We understand the reviewer’s concern. In addition to the validation using data from 10 head-and-neck cancer patients, the model was initially validated against two independent murine studies from the literature. The first study involved murine HPV-positive head and neck tumors, comprising one control group and one treatment group. The second study focused on murine prostate cancer models, including one control group and four treatment groups. Therefore, the model has been validated using both head and neck cancer cases (pre-clinical and clinical data) as well as prostate cancer cases (pre-clinical data). Nevertheless, we have revised the manuscript to explicitly address this limitation in the “Discussion” section (page 30).

*******Results***

*What are the characteristics of the patients in whom the model was tested for prostate cancer? Which cohort was used? Providing answers to these questions would contribute to a more precise interpretation of the findings.*

We thank the reviewer for the valuable questions. As noted earlier, tumor volume measurements during radiotherapy are rarely available, as they are not part of routine clinical practice. This is especially true for prostate cancer, where treatment response is typically monitored via PSA levels rather than direct volumetric assessment. Therefore, due to the lack of detailed clinical data our simulations were not based on a specific cohort of prostate cancer patients.

As a result, parameters not reported in the literature were estimated based on our daily clinical experience treating both prostate and head and neck cancer patients at the hospital. Clinically, prostate tumors are observed to grow much more slowly and exhibit less aggressive behavior than head and neck tumors. To capture this biological difference, we reduced the transition rate parameter $k_{M}$ to one-eighth of the value used for head and neck cancer. Additionally, the clearance rate $C_{r}$ was assigned the same value as in patients HN06 and HN10 to maintain consistency across simulations and facilitate comparative analysis.

Even if prostate tumor volume measurements during radiotherapy were available, only the clearance rate $C_{r}$ could be fitted to clinical data. Fitting the transition rate $k_{M}$ would require data from untreated patients, which is not feasible in a clinical setting where all patients receive treatment.

*In Figures 9A, 10A, 10C, and 11A, 11C, despite slight differences when considering treatment durations, no distinct separation is observed in the graphs. The interpretations regarding these figures might be overestimated. In contrast, in Figures 9B, 9C, 10B, and 11B, cell survival rates in radioresistant conditions appear to be significantly affected at a clinically meaningful level.*

We thank the reviewer for this insightful observation. We agree that the differences in Figures 9A, 10A, 10C, and 11A, 11C are subtle and may not support strong conclusions. Accordingly, we have revised the corresponding interpretations in the “Results” (pages 25-27) section of the manuscript to more accurately reflect the observed data and to avoid overestimation. We also emphasize the more clinically meaningful findings from Figures 9B, 9C, 10B, and 11B, as suggested.

# Reviewer #4

**Summary**

*This study develops a mathematical model to explore how tumor perfusion/oxygenation and radiotherapy schedules influence treatment efficacy. The model integrates cell cycle dynamics, radiation effects via the Linear Quadratic model, oxygen diffusion, and tumor biomechanics. Validated with preclinical (murine head/neck and prostate tumors) and clinical (head/neck cancer patients) data, the work highlights that increased perfusion enhances radiation effectiveness, particularly in radioresistant tumors. The model also suggests hyperfractionation benefits aggressive tumors, while stereotactic body radiotherapy (SBRT) achieves rapid tumor control in prostate cancer.*

**Strengths**

*Novelty: First continuous model to combine radiotherapy schedules, perfusion, and oxygenation. Provides mechanistic insights into microenvironment modulation.*

*Comprehensive Validation: Robust validation across preclinical (murine) and clinical (human) datasets, demonstrating generalizability.*

*Clinical Relevance: Highlights the importance of vascular normalization strategies and optimal fractionation (e.g., SBRT for prostate cancer).*

*Methodological Rigor: Detailed integration of cell cycle phases, oxygen dynamics, and biomechanical tumor growth.*

We thank the Reviewer for appreciating the novelty of our work and for the constructive comments.

**Weaknesses**

*Parameterization Concerns: Some parameters were fitted due to limited data, risking overfitting.*

We understand the reviewer’s concern. To minimize the risk of overfitting during model validation against preclinical cases, only one parameter out of 29 was adjusted per simulation, with all others held constant. It is also worth mentioning that for the murine prostate cancer models, we fitted only the transition rate $k_{M}$ on the control group and only the clearance rate $C_{r}$ on the 3x5 Gy case. For the rest of the 4 cases/treatment groups, no parameters were fitted – all values of model parameters were kept constant and the model provided a good fit to the data. This approach demonstrates the model’s robustness and ability to generalize across different treatment conditions with minimal parameter tuning.

*Radiosensitivity parameters (α/β) for prostate cancer were assumed uniform across cell phases without explicit validation.*

The reviewer is right. It is known that cell radiosensitivity varies significantly throughout the cell cycle. Consequently, radiosensitivity parameters $a$ and $\beta$ are expected to vary phase by phase. Despite this, clinical studies typically aim to determine the $a/\beta$ ratio for each tumor type, often overlooking the intra-cycle variation radiosensitivity. Having said that, due to the limited availability of phase-specific data, we adopted the simplifying assumption of uniform radiosensitivity across all cell cycle phases for the prostate cancer case. Importantly, this is addressed in the “Discussion” section (page 29) as a limitation of our model.

*Simplified Assumptions: Isotropic tumor growth neglects heterogeneity seen in real tumors. Normal tissue toxicity was omitted, limiting clinical applicability.*

The reviewer is right. These limitations are acknowledged and discussed in detail in the “Discussion” section (pages 29-30). Specifically, we noted that future work will incorporate anisotropic proliferation by assigning distinct growth stretch ratios $\lambda_{g}$ to each spatial direction. Furthermore, to address the omission of normal tissue toxicity, we emphasized the need for future studies to integrate the normal tissue complication probability (NTCP) into the modeling framework.

*Limited Clinical Data: Small human sample sizes (e.g., 10 head/neck patients) may reduce statistical power.*

The reviewer is right. As stated before, tumor volume measurements are rarely incorporated into radiotherapy studies, as volumetric assessment is not a routine clinical practice. In prostate cancer, for example, the entire gland is typically irradiated due to challenges in localizing malignant areas, making volume reduction an unreliable indicator of response. In fact, the prostate may enlarge during treatment due to radiation-induced edema. Instead, treatment efficacy is commonly assessed via prostate-specific antigen (PSA) levels rather than direct volume changes.

As a result, due to the limited availability of clinical data, the model was initially validated using two independent murine studies from the literature. The first study involved HPV-positive head and neck tumors in mice, with one control group and one treatment group. The second study focused on murine prostate cancer models and included one control group and four treatment groups.

*Lack of Sensitivity Analysis: Unclear how parameter uncertainties affect predictions.*

We thank the reviewer for the valuable suggestion. In response, we have conducted a sensitivity analysis to evaluate the impact of parameter uncertainties on model predictions. We focused the analysis on the parameters discussed more in our work and these are: the parameter $a$ of the Linear Quadratic model, the transition rate $k_{M}$ and the clearance rate $C_{r}$. The results of this analysis are now included in the Supplementary Material, Supplementary Figures **S11** to **S13**.

**Recommendations**

*Parameter Justification: Clarify how fitted parameters were derived and assess robustness via sensitivity analysis.*

We thank the reviewer for the valuable suggestion. For each parameter fitting we first computed the mean of the experimental tumor volume data, denoted as $y_{mean}$, from either preclinical or clinical studies. The total sum of squares ${SS}_{total}$ was then calculated using:

$${SS}_{total}=\sum\left( y_{experimental}-y_{mean} \right)^{2}$$

where $y_{experimental}$ represents the measured experimental tumor volumes at various time points during radiotherapy. Next, we calculated the residual sum of squares ${SS}_{residual}$ as:

$${SS}_{residual}=\sum\left( y_{experimental}-y_{simulated} \right)^{2}$$

where $y_{simulated}$ denotes the corresponding tumor volumes generated by the model. The coefficient of determination $R^{2}$ was then calculated as:

$$R^{2}=1-\frac{{SS}_{residual}}{{SS}_{total}}$$

An $R^{2}$ value of 1 indicates a perfect fit between the simulated and experimental data. Accordingly, the transition rate $k_{M}$ and the clearance rate $C_{r}$ were adjusted to the control and treated groups respectively, to maximize $R^{2}$, aiming for values as close to 1 as possible. A brief summary of this is included in the validation section of the model (page 18).

With respect to the sensitivity analysis, we kindly refer the reviewer to our response above.

*Address Model Limitations: Discuss implications of isotropic growth and absence of normal tissue toxicity on clinical translation.*

We thank the reviewer for the valuable comment. In response, we have expanded the “Discussion” section (pages 29-30) to address the implications of assuming isotropic tumor growth and the omission of normal tissue toxicity.

*Expand Clinical Context:*

*Discuss practical strategies to modulate perfusion (e.g., anti-angiogenics, vascular normalization).*

We thank the reviewer for the valuable input. In the “Discussion” section (pages 30-31), we included a paragraph on promoting vascular and stroma normalization as a strategy to enhance the effectiveness of radiotherapy.

*Compare model predictions with clinical trials on hypofractionation/SBRT.*

We appreciate the reviewer’s suggestion. As previously noted, clinical radiotherapy studies rarely include direct measurements of tumor volume or cancer cell survival, as these are not part of standard clinical practice. Instead, clinical outcomes are typically assessed in terms of local-regional control or recurrence rates over extended follow-up periods. Due to this discrepancy in outcome measures, a direct comparison between our model predictions and clinical trial data is not currently feasible.

*Include Sensitivity Analysis: Identify parameters most critical to outcomes to guide future experimental validation.*

We thank the reviewer for the comment and kindly refer to our response above. *Enhance Discussion:*

*Contrast findings with prior models (e.g., discrete vs. continuous approaches).*

We appreciate the reviewer’s suggestion. To the best of our knowledge, this is the first continuous mathematical model that explores different radiotherapy schedules while accounting for dynamic changes in perfusion and oxygenation. Given the lack of similar models in literature, direct comparisons are currently not feasible.

*Explore how tumor subtype/genetic heterogeneity might influence results.*

We appreciate the reviewer’s valuable suggestion. However, examining the influence of tumor subtype and genetic heterogeneity falls outside the scope of this study. Our focus is primarily on the general modeling of radiotherapy schedules and their effects on tumor response, with an emphasis on variations in radiosensitivity and oxygenation, rather than tumor-specific genetic factors.

**Conclusions**

*This manuscript presents a sophisticated model advancing our understanding of radiotherapy optimization through microenvironment modulation. While the methodology is rigorous and findings clinically relevant, addressing parameterization transparency and model limitations would strengthen impact. With revisions, this work could inform personalized radiotherapy protocols and combination therapies targeting tumor perfusion.*

We thank the reviewer for the comment. We revised the “Model validation with preclinical data” section (page 18) to better explain the fitting process. In addition, the “Discussion” section (pages 29-30) has been expanded to outline key assumptions and limitations of the model. These include the use of uniform $a$ and $\beta$ values due to limited data, simplified tumor geometry and isotropic proliferation. Future work will address these by incorporating anisotropic growth, patient-specific properties, immune response dynamics and normal tissue complication probability (NTCP), aiming to enhance the model’s accuracy and clinical relevance.

*Decision: Minor Revisions (Address parameter justification, sensitivity analysis, and clinical applicability).*

We thank the reviewer for their valuable feedback. Please see our response in the previous comment.

*Technical Soundness and Data Support for Conclusions: The manuscript demonstrates moderate technical soundness with notable strengths and limitations:*

*Model Validity:*

*Strengths:*

*The integration of cell cycle dynamics, oxygen diffusion, and biomechanical growth into a continuous model is innovative and methodologically rigorous.*

*Validation across preclinical (murine) and clinical (head/neck cancer) datasets strengthens the model’s generalizability.*

*Limitations:*

*Parameter Overfitting: Critical parameters were fitted to match limited experimental data, raising concerns about model specificity. For example, cell cycle transition rate was adjusted differently for murine vs. human data without biological justification.*

We acknowledge the reviewer’s concern. To minimize the risk of overfitting during model validation against preclinical cases, only one parameter out of 29 was adjusted per simulation, with all others held constant. Specifically, for the murine prostate cancer models, we fitted only the transition rate $k_{M}$ on the control group and only the clearance rate $C_{r}$ on the 3x5 Gy case. For the rest of the cases, no parameters were fitted – all values were kept constant. This approach demonstrates the model’s robustness and ability to generalize across different treatment conditions with minimal parameter tuning. Regarding the example raised by the reviewer concerning the adjustment of the cell cycle transition rate for murine and human data, this parameter was calibrated to best fit the control groups in murine studies and the treated groups in human studies. The goal was to maximize the coefficient of determination $R^{2}$, aiming for values as close to 1 as possible. This adjustment was made to improve model fit and was not based on a specific biological rationale.

*Radiosensitivity Assumptions: Uniform \alpha/\betaα/β ratios across cell phases (e.g., prostate cancer) lack experimental validation. Prior studies (e.g., Brenner et al., 1999) suggest cell cycle-dependent radiosensitivity, which is not addressed.*

The reviewer is right. It is known that cell radiosensitivity varies significantly throughout the cell cycle. Consequently, radiosensitivity parameters $a$ and $\beta$ are expected to vary phase by phase. Despite this, clinical studies typically aim to determine the $a/\beta$ ratio for each tumor type, often overlooking the intra-cycle variation radiosensitivity. Having said that, due to the limited availability of phase-specific data, we adopted the simplifying assumption of uniform radiosensitivity across all cell cycle phases for the prostate cancer case. Importantly, this is addressed in the “Discussion” section as a limitation of our model.

*Data Support:*

*Preclinical Data: Murine tumor growth curves (Figs 3–4) align with model predictions (R^2 > 0.85), supporting the model’s ability to replicate in vivo dynamics.*

*Clinical Data: Human head/neck tumor volume changes (Figs 5–6) show qualitative agreement but lack statistical significance due to small sample sizes (n=10). Larger cohorts are needed to confirm clinical relevance.*

We appreciate the reviewer’s suggestion. However, as previously noted, clinical radiotherapy studies rarely report direct measurements of tumor volume or cancer cell survival, as these are not standard endpoints in clinical practice. Instead, outcomes are typically assessed through metrics such as local-regional control or recurrence rates over long-term follow-up. This limitation in available clinical data constrains the ability to perform statistically robust comparisons, particularly with small sample sizes.

*Key Conclusion: The claim that “increased perfusion enhances radiotherapy efficacy in radioresistant tumors” is supported by simulations (Figs 9–11) but requires in vivo validation (e.g., hypoxia imaging or perfusion biomarkers).*

We thank the reviewer for the valuable feedback. We agree that in vivo validation—such as through hypoxia imaging or perfusion biomarkers—would strengthen the conclusions. However, performing in vivo experiments is beyond the scope of the current study, which is focused on computational modeling and simulation-based insights. To support the biological plausibility of our findings, we include in the “Discussion” section references to relevant studies that have demonstrated associations between decreased perfusion, tumor hypoxia, and reduced radiotherapy efficacy. (page 30)

**Statistical Analysis and Rigor**

*The statistical analysis is partially rigorous:*

*Model Validation:*

*R^2 values are reported for preclinical data fits, but confidence intervals or error margins are missing.*

We acknowledge the reviewer’s concern. In this case, our model is deterministic, meaning that for a given set of input parameters and initial conditions, the outcome is always the same—there is no inherent randomness in the simulation process. As such, running the model multiple times with the same inputs will yield identical results. Therefore, confidence intervals, which are typically used to express the uncertainty due to stochastic variability or sampling error, are not applicable in this context.

*No sensitivity analysis is performed to quantify parameter uncertainty (e.g., Monte Carlo methods), which is critical for deterministic models.*

We thank the reviewer for their valuable feedback. Please see our response in the previous comment.

*Clinical Data:*

*Volumetric tumor changes in patients are described descriptively (no p-values or effect sizes). Statistical tests (e.g., paired t-tests between predicted vs. observed volumes) are absent.*

We thank the reviewer for the valuable feedback. In response, we have added Bland-Altman plots to the supplementary material for the clinical data, as suggested by the reviewer on the upcoming recommendation. These plots provide a complementary assessment to the $R^{2}$ values we reported for each model fitting, offering an additional perspective on the agreement between predicted and observed tumor volumes.

*Recommendation:*

*Include sensitivity analysis to identify high-impact parameters.*

We thank the reviewer for their valuable feedback. Please see our response in the previous comment.

*Apply statistical tests (e.g., Bland-Altman plots) to quantify model-clinical data discrepancies (discrepancies σημαίνει αποκλίσεις).*

**Data Availability**

*The authors state: “All relevant data are within the manuscript and its Supporting Information files.”*

*However:*

*Code/Model Availability: No mention of computational code or model scripts (e.g., COMSOL files) being shared, limiting reproducibility.*

The reviewer is right. We added a “Data Availability” section stating that the full COMSOL Multiphysics model report is included in the Supporting Information. It contains comprehensive simulation details necessary for replication or further analysis.
*Raw Simulation Data: Figures show processed outputs, but raw simulation datasets (e.g., oxygen concentration gradients, stress maps) are not provided.*

We thank the reviewer for the comment regarding the availability of raw simulation data. Due to the complexity of the biophysical model, which incorporates numerous interacting variables, and the extensive simulation protocol—three different radiotherapy schedules, each combined with three tumor radiosensitivity scenarios and two functional vascular density conditions—the total volume of raw simulation output (e.g., oxygen gradients, stress maps) is substantial to be uploaded and it is not required by the journal.

As such, including all raw data within the manuscript or supplementary materials would be difficult to manage effectively. However, we are happy to provide specific datasets upon request, or alternatively, we can share the COMSOL model files should the reviewer wish to explore or reproduce the simulations independently.

*Recommendation:
Deposit code and raw data in public repositories (e.g., GitHub, Zenodo) with DOIs.*

Please see our response to the previous comment.

**Clarity and Language**

*The manuscript is largely intelligible but has minor issues:*

*Technical Jargon: Terms like “multiplicative decomposition of deformation gradient tensor” (p. 12) are inadequately explained for non-specialists.*

We thank the reviewer for the comment. We modified the manuscript to provide a clearer explanation of the "multiplicative decomposition of the deformation gradient tensor" in more accessible terms (page 12).

*Figure Clarity:*

*Fig 9–11 legends lack explicit definitions of “low” vs. “high” vascular density thresholds.*

We thank the reviewer for the comment. We have updated the figure legends for Figures 9–11 to explicitly define the thresholds for "low" and "high" vascular density, as requested.

*Axis labels in Figs 3–6 are too small for readability.*

We thank the reviewer for pointing this out. As figures are required to be submitted separately and are formatted according to the journal’s guidelines during the review process, we are unsure of the exact display size seen by the reviewer. Nevertheless, we have reviewed Figures 3–6 and increased the font size of the axis labels to improve readability. We trust this will enhance clarity in the final version.

*Grammar: Occasional errors (e.g., “submits” instead of “undergoes apoptosis” on p. 4) require proofreading.*

We thank the reviewer for the comment. In response, we have updated the manuscript to incorporate the suggested revision.

*Recommendation:*

*Simplify biomechanical terminology in the Methods section.*

*Revise figures to meet PLOS ONE formatting guidelines (e.g., font size ≥ 8pt).*

We thank the Reviewer. We have addressed these issues in the revised manuscript.

**Ethical Considerations**

*Dual Publication: No evidence of duplicate submission.*

*Ethics Compliance: The human study (Barker et al., 2004) cites institutional approval, but patient consent details are omitted.*

We thank the reviewer for the comment. We would like to clarify that we were not involved in the Barker et al. (2004) study and did not have access to the original patient consent details. Our use of this study was solely to validate our model using their published data. We relied on the citation provided in the original publication, which includes the necessary ethical approval information.

*Funding Transparency: ERC and EU funding are appropriately disclosed.*

**Overall Recommendation**

*Minor Revisions prior to publication:*

*Address parameter justification and sensitivity analysis.*

We thank the reviewer for the comment. In response, we have included a detailed justification for the parameters used in our model, as well as a comprehensive sensitivity analysis, which is now provided in the manuscript and supplementary material.

*Share computational code and raw simulation data.*

The reviewer is right. We added a “Data Availability” section stating that the full COMSOL Multiphysics model report is included in the Supporting Information. It contains comprehensive simulation details necessary for replication or further analysis. Regarding the raw simulation data, we are happy to provide specific datasets upon request, or alternatively, we can share the COMSOL model files should the reviewer wish to explore or reproduce the simulations independently.

*Improve statistical reporting (confidence intervals, error margins).*

We thank the reviewer for the suggestion. Since our model is deterministic, running the model multiple times with the same inputs will yield identical results. Therefore, confidence intervals, which are typically used to express the uncertainty due to stochastic variability or sampling error, are not applicable in this context. However, in response to the reviewer’s previous recommendation, we have added Bland-Altman plots for the clinical data to the supplementary material. These plots provide a complementary assessment to the $R^{2}$ values we reported for each model fitting, offering an additional perspective on the agreement between predicted and observed tumor volumes.

*Simplify technical language and enhance figure clarity.*

We thank the reviewer for the comment. In response, we have simplified the technical language as much as possible throughout the manuscript and made revisions to enhance the clarity of the figures.

*The study provides valuable insights into radiotherapy optimization but requires stronger statistical rigor and transparency to fulfill reproducibility standards.*
