# Supplementary material for: The impact of tumor microenvironment and treatment schedule on the effectiveness of radiation therapy
Source: PLoS One. 2025 Sep 17;20(9):e0331509. doi: 10.1371/journal.pone.0331509 (PMC12443294; doi:10.1371/journal.pone.0331509)
Supplement: Attachment — Submitted filename: Response_to_Reviewers_auresp_2.docx [file pone.0331509.s017.docx]

Editor in Chief, PLOS ONE

August 12, 2025

Dear Editor,

We are pleased to submit our revised manuscript entitled “The impact of tumor microenvironment and treatment schedule on the effectiveness of radiation therapy” for publication in PLOS ONE*.*

We thank you for your thoughtful and helpful comments that helped us revise our manuscript. As summarized in the attached point-by-point response, we have addressed all of your comments.

All co-authors have had the opportunity to review the manuscript and approved this submission.

Please find below our responses and the actions taken to address each of the comments:

1. *The prostate cancer parameters lack clinical validation: The authors state that the prostate cancer parameters (such as kM and Cr) were set based on "clinical experience" due to the "lack of clinical data" (lines 447-451). This significantly undermines the credibility of the conclusion. It is recommended to provide additional literature support (such as citing studies on the proliferation rate of prostate cancer cells). Or clearly label this as an exploratory analysis and emphasize this limitation in the discussion (such as lines 616-617). Consider using alternative indicators (such as PSA kinetics) for indirect verification.*

We appreciate the editor’s suggestion. In the Discussion section we have added the following: “Given this limitation, it is important to emphasize that the prostate cancer modeling presented here should be regarded as exploratory. While this approach allows for reasonable simulations aligned with daily clinical observations, it does not replace the need for rigorous clinical validation. As such, the results for prostate cancer should be interpreted with caution, and future work should prioritize parameter estimation based on direct clinical measurements or PSA-derived tumor kinetics, as data becomes available.”

2. *Immune response and tumor heterogeneity: The model did not incorporate the immune microenvironment (lines 596-599) and tumor spatial heterogeneity (such as dynamic changes in hypoxic regions). It is recommended to quantify the potential impact of these factors on the results in the discussion (for example: immune exhaustion may reduce the predicted cell killing rate by the model). Cite relevant studies to illustrate its significance (such as the impact of tumor-associated fibroblasts on perfusion).*

We thank the editor for the valuable input. In the Discussion section we have added the following: “As a result, the combination of radiotherapy and immunotherapy does not consistently produce a synergistic effect. Taking into account mechanisms related to anti-tumor immune responses will likely affect the predicted by the model cell killing rate. Excluding these effects may lead to either an over- or underestimation of treatment efficacy, depending on tumor's immune profile. Incorporating such mechanisms could improve the accuracy of predictions, particularly in cases where immune dynamics play a significant role in patient outcomes.”

3. *Figures and Data: Figure 7: The unit for the x-axis is missing (at line 437). The unit for the time axis in the figure (which should be "Days") is not indicated. This affects the interpretation. It is recommended to add the unit for the x-axis in the revised version. The statistical significance of Figure 9-11: The difference between the high/low vascular density curves does not provide statistical test results (such as p-values). It is recommended to supplement the statistical indicators of sensitivity analysis (such as confidence intervals) or explain that this is a deterministic model simulation, focusing on the trend rather than the statistical difference.*

We thank the editor for the comments. We have added the unit for the time axis in Figure 7. In addition, in the Results section we have added the following: “It should be noted that the results shown in Figs 9–11 are based on deterministic model simulations. As such, these figures are intended to highlight how the system responds to changes in vascular density, focusing on overall trends rather than statistically significant differences.”

4. *Terminology inconsistency: "radioresistance" appears in the text with multiple spellings (radio-resistance/radioresistant, on lines 121/485). It is recommended to standardize it as "radioresistance".*

We appreciate the editor’s valuable input. We use the standardized term “radioresistance” throughout the manuscript.

5. *Clinical translation suggestion: There was no in-depth discussion on how to utilize the model to guide clinical strategies (such as the timing of combining vascular normalization drugs). It is recommended to add specific suggestions at the end of the discussion (for example: "Based on the model results, we suggest initiating anti-vascular treatment 2 weeks before SBRT to maximize oxygenation improvement").*

We thank the editor for the suggestion. In the Discussion section we have added the following: “Based on pertinent clinical studies, even one week of pre-treatment with a mechanotherapeutic, it is sufficient to normalize the tumor stroma of pancreatic tumors and sarcomas. Anti-angiogenic treatment to normalize the tumor vasculature is time- and dose-dependent and not easy to control because elongated treatment or high dose can cause excessive vessel pruning and thus, reduced perfusion. Therefore, close monitoring of vessel functionality during anti-angiogenic treatment with medical imaging methods is required.”

6. *Lack of toxicity assessment: Ignoring the probability of normal tissue complications (NTCP) is a significant limitation (lines 603-608), but its impact has not been quantified. It is recommended to cite clinical data to illustrate the toxicity differences (such as the urinary toxicity risk of SBRT [14]).*

We appreciate the editor’s comment. In the Discussion section, alongside toxicity data for prostate SBRT, we have now included toxicity data for head and neck cancer to emphasize the importance of considering normal tissue complication probability.

7. *Abbreviations: When an abbreviation is first used, the full term must be indicated (for example, "SBRT" should be written as "Stereotactic Body Radiotherapy (SBRT)" in the abstract when it is first mentioned).*

We thank the editor for the suggestion. We have revised the manuscript to ensure that all abbreviations are defined in full upon first use, including changing “SBRT” to “Stereotactic Body Radiotherapy (SBRT)” in the abstract and other relevant sections.

8. *Introduction: The global cancer epidemiological data (lines 66-71) can be summarized in 1-2 sentences, highlighting the cancer types related to radiotherapy.*

We appreciate the editor’s comment. We have revised the Introduction to condense the global cancer epidemiological data into two sentences, focusing on the cancer types most relevant to radiotherapy.

9. *References: Supplement the research on new radiotherapy protocols and tumor microenvironment (such as immunotherapy combined strategies) for the years 2023-2024.*

We thank the editor for this valuable suggestion. In the Discussion section we have added the following: “While this study focuses on stereotactic body radiotherapy, hypofractionated and conventionally fractionated treatments, emerging radiotherapy methods are rapidly advancing the field. Techniques like Adaptive Radioherapy (ART) and Surface-Guided Radiotherapy (SGRT) enhance precision by adjusting to daily anatomical changes and tracking patient positioning in real time. Alongside AI-driven imaging, MR-LINACs, particle therapy and emerging approaches such as FLASH radiotherapy and radioimmunotherapy, these innovations offer more personalized, targeted treatments that improve tumor control while minimizing damage to healthy tissue.”

We have also updated our references to include recent studies from 2023–2024, with a primary focus on the tumor microenvironment. Notable examples include:

Kalli M, Stylianopoulos T. Toward innovative approaches for exploring the mechanically regulated tumor-immune microenvironment. APL Bioeng 2024;8:011501. <https://doi.org/10.1063/5.0183302>.

Mpekris F, Papaphilippou PCh, Panagi M, Voutouri C, Michael C, Charalambous A, et al. Pirfenidone-Loaded Polymeric Micelles as an Effective Mechanotherapeutic to Potentiate Immunotherapy in Mouse Tumor Models. ACS Nano 2023;17:24654–67. <https://doi.org/10.1021/acsnano.3c03305>.

Kalli M, Mpekris F, Charalambous A, Michael C, Stylianou C, Voutouri C, et al. Mechanical forces inducing oxaliplatin resistance in pancreatic cancer can be targeted by autophagy inhibition. Commun Biol 2024;7:1581. <https://doi.org/10.1038/s42003-024-07268-1>.

Kalli M, Poskus MD, Stylianopoulos T, Zervantonakis IK. Beyond matrix stiffness: targeting force-induced cancer drug resistance. Trends Cancer 2023;9:937–54. <https://doi.org/10.1016/j.trecan.2023.07.006>.

Panagi M, Mpekris F, Voutouri C, Hadjigeorgiou AG, Symeonidou C, Porfyriou E, et al. Stabilizing Tumor-Resident Mast Cells Restores T-Cell Infiltration and Sensitizes Sarcomas to PD-L1 Inhibition. Clin Cancer Res 2024;30:2582–97. https://doi.org/10.1158/1078-0432.CCR-24-0246.

Thank you for your consideration of this work and please do not hesitate to reach out if you require any additional information.

Sincerely,

Prof. Triantafyllos Stylianopoulos

ERC Consolidator Grant Fellow, Department of Mechanical Engineering, University of Cyprus, Nicosia, Cyprus

Email: tstylian@ucy.ac1.cy
